# Vital Signs Detection by a 24 GHz Radar Sensor Operating in Linear/Circular Polarization in the Presence of Environmental Clutter

**DOI:** 10.3390/s26154663

**Published:** 2026-07-23

**Authors:** Renato Cicchetti, Stefano Pisa, Emanuele Piuzzi, Matteo Pistillucci, Angelo Forte, Orlandino Testa

**Affiliations:** Department of Information Engineering, Electronics and Telecommunications, Sapienza University of Rome, Via Eudossiana 18, 00184 Rome, Italy; renato.cicchetti@uniroma1.it (R.C.); stefano.pisa@uniroma1.it (S.P.); matteo.pistillucci@uniroma1.it (M.P.); a.forte@uniroma1.it (A.F.); orlandino.testa@uniroma1.it (O.T.)

**Keywords:** polarization converters, radar sensors, uniaxial tensor, vital signs detection

## Abstract

A 24 GHz radar, useful for remote vital signs monitoring, operating in linear/circular polarization (LP/CP), was developed to analyze vital signs detection performance in the presence of environmental clutter. The radar system, equipped with horn antennas with and without suitable LP-CP field polarization converters, is designed for monitoring respiratory and cardiac activity in domestic and hospital environments. A geometric optics (GO) model is introduced to show the advantages of circularly polarized (CP) radar sensors over linearly polarized (LP) ones in discriminating vital signals in scenarios where vibrating metallic panels simulate environmental disturbance (worst case). An analytical model, numerical computations, and experimental investigations highlighted the robustness of CP radar sensors in detecting vital signs in the presence of environmental clutter.

## 1. Introduction

The last ten years have seen significant developments in automotive radar systems [1], proximity sensors [2] and remote vital signs monitoring [3]. In this context, radar sensors can facilitate the non-invasive monitoring of people in a non-collaborative state (sleeping elderly, newborns, burn victims, etc.), where measurements without sensor–individual contact are preferable. Similar systems could be useful as support devices for the continuous monitoring of drivers of road vehicles to detect critical driving states (drowsiness, tiredness, etc.), thus increasing road safety [4]. Compared to other vital signs monitoring systems, such as those based on optical recording or video-based sensors [5,6,7,8], microwave and millimeter-wave radar sensors exhibit reduced design and production costs and competitive performance since they are able to carry out detections through clothing (privacy protection), thus being scarcely influenced by ambient lighting or environmental conditions [9,10]. These sensors exploit phase variations in the signal reflected by the subject for monitoring the cardiorespiratory rhythm generated by the small movements of the human chest wall caused by the heartbeat or respiratory activity (on the order of 0.3 mm and 6 mm for cardiac and respiratory activity, respectively) [11]. The relationship between phase variation and physical displacement of the chest wall is used to extract information related to vital signs (heart and respiratory rate, apnea, cardiac arrest, etc.). For example, the relationship between phase variation and target displacement is about 1 rad/mm (radar sensor sensitivity) at 24 GHz [12], while higher sensitivities can be obtained with radar sensors operating at higher frequencies [13]. Although radar sensors operating at higher frequencies have greater sensitivity, they may present potential issues in detecting the phase of the received signal when they are used for respiratory activity detection, since the amplitude of the chest movement is significantly greater than that of the R.F. signal wavelength [14]. In this context, 24 GHz radar sensors represent an excellent compromise because the R.F. wavelength is close to the displacement induced by both respiratory and cardiac activity, thus ensuring good detection of vital signs.

As usually happens in radio communication systems, microwave and millimeter-wave radar sensors are significantly affected by interference due to environmental multipath [15]. This phenomenon is responsible for the arrival at the Rx antenna of several RF field contributions linked to the different optical paths (each having a different phase delay), which can cause errors in the detection of the vital signals of the monitored subject. In this context, cardiac activity detection is more affected by multipath interference, since the level of electromagnetic signals reflected from the human chest due to the heartbeat is generally an order of magnitude weaker than those due to respiratory activity [16,17,18]. Therefore, to increase the signal-to-noise ratio (SNR) at the radar sensor output, it is strongly recommended to implement all the necessary hardware and software strategies. Although signal processing software plays an important role in increasing SNR both in radar [10] and optical sensors [19,20], hardware solutions can further improve the quality of the received signal (up to 12–15 dB) by properly selecting the modulation technique [single frequency continuous wave (SFCW), frequency modulated continuous wave (FMCW), etc.], the Tx-Rx antenna system [single input single output (SISO), single input multiple output (SIMO), multiple input multiple output (MIMO)], as well as the polarization of the electromagnetic field (horizontal, vertical, circular, etc.) [10].

For these reasons, in addition to widespread applications in automotive and proximity micrometer sensors, research is currently underway to identify vital signals by means of radar sensors based on architectures, antennas, field polarizations, as well as signal modulations that best meet the SNR increase requirements for systems operating in noisy environments.

For the above-mentioned applications, linear polarization has been mainly used because it is more cost-effective and simpler to implement, while only a few examples of radar sensors operating in circular polarization are currently available in the literature [21,22,23]. A circularly polarized ultra-wideband (UWB) radar sensor equipped with a sequentially rotated Vivaldi antenna array was presented in [21]. This solution, employing three hybrid couplers to excite the four orthogonal to each other LP dual elliptically tapered antipodal slot Vivaldi antennas, was chosen to ensure a higher level of the received field scattered by the target excited by the radar sensor. Although CP-UWB radar sensors are less sensitive to variations in the monitored subject’s radar cross-section (RCS) [21], they are more vulnerable to environmental electromagnetic clutter. In [22], an orthogonally excited square patch array, fed by a modified ring-shaped quadrature hybrid coupler, was used to realize a Doppler radar sensor operating at 2.4 GHz. The single dual-CP antenna achieves 280% size reduction compared to a traditional two-linear-polarization patch antenna array with the same isolation level. Finally, a short-range Doppler sensor operating at 5.8 GHz based on a sequentially rotated square patch array, excited by a four-way power divider, obtained by a cascade of two Wilkinson dividers, was presented in [23]. The gain of the sensor antennas is about 6 dBi with high TX–RX electrical isolation exceeding 60 dB at the operating frequency.

Although the systems presented in [21,22,23] describe radar sensors for vital signs operating in CP, none of them is aimed at verifying the robustness of such field polarization with respect to environmental clutter. To this end, in this paper, a radar sensor for remote monitoring of vital activities, suitable for operating in both linear and circular polarization in highly controlled operating contexts, is presented. Both numerical and experimental investigations were performed to verify the degree of robustness and sensitivity of the radar system with respect to environmental clutter when operating in circular polarization.

The article is divided into six sections. In Section 2, the radar sensor architecture is presented; in Section 3, the design of the LP-CP dielectric polarization converter employed in the radar sensor is described; in Section 4, the GO model used to justify the robustness of the CP in the presence of environmental clutter is illustrated; in Section 5, the numerical and experimental results are presented; and in Section 6, the conclusions are drawn.

## 2. Radar Sensor Architecture

**Figure 1:**
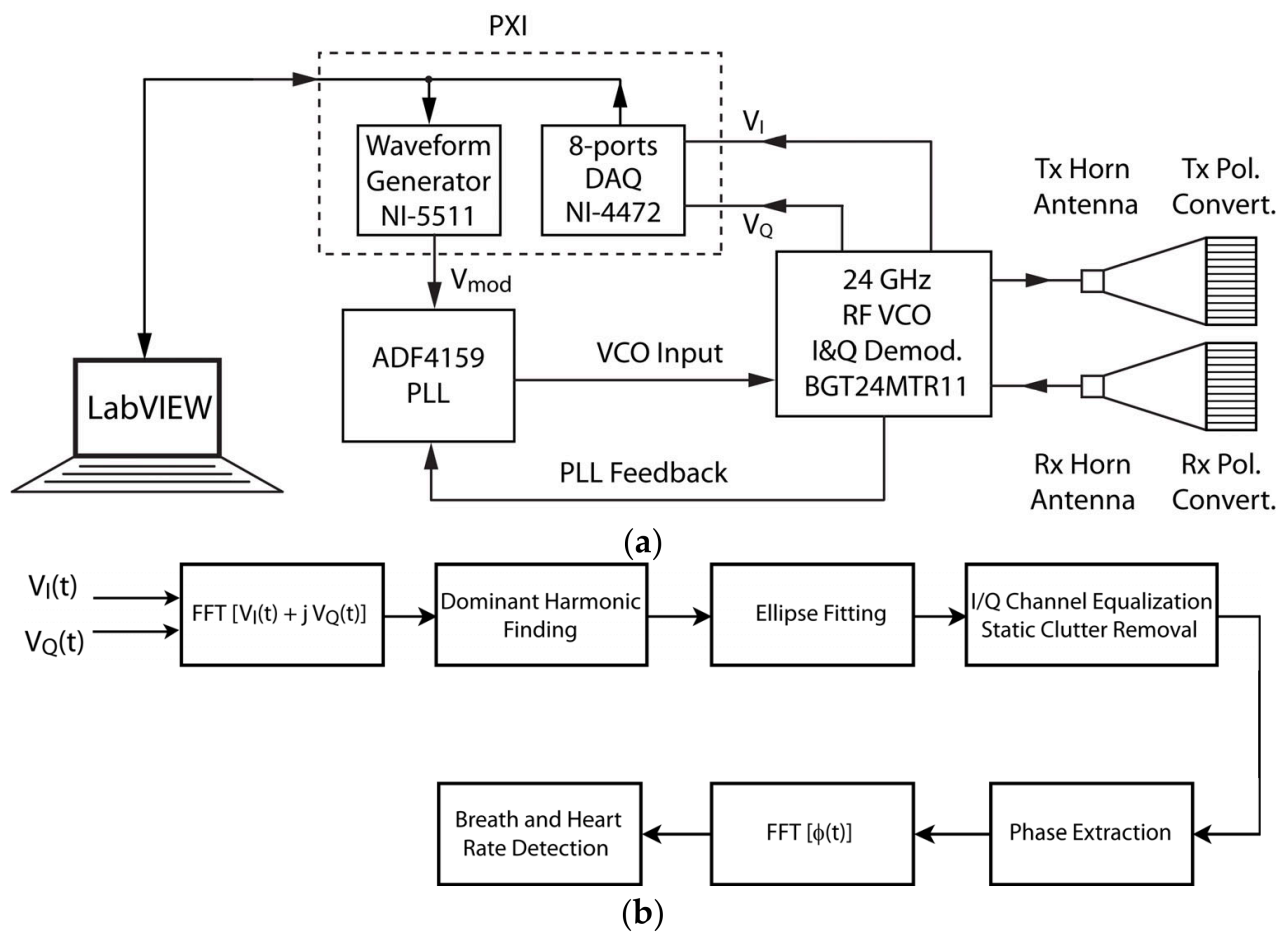
Block diagrams: (**a**) of the 24 GHz SFMCW radar with Tx and Rx antennas equipped with LP-CP converters, (**b**) of the implemented signal processing.

The radar operating at 24 GHz, used for the experimental activities, consists of an Infineon BGT24MTR11 module (Infineon Technologies, Munich, Germany) integrated with a ADF4159 PLL board (Analog Devices, Norwood, MA, USA) and two high-gain Pasternack horn antennas (PE9852/2F-15) (Pasternack, Irvine, CA, USA) employed as transmitting (Tx) and receiving (Rx) antennas. The radar system is controlled by an NI PXI 1042 device (National Instruments, Austin, TX, USA) equipped with a NI-5411 waveform generator and an 8-channel NI-4472 ADC acquisition board, managed through a LabVIEW program (see Figure 1a).

The sinusoidal signal, used for FM modulation of the RF carrier, was generated by using a periodic control signal produced by the NI-5411 board to excite the PLL trigger port. The resulting SFMCW signal exhibits a frequency band in the 24.00–24.25 GHz range with steep roll-off, allowing the radar to achieve a very high phase sensitivity (about 1.0 rad/mm) due to the low phase noise of the RF circuitry (note that high phase noise levels can significantly reduce the sensor resolution) and the high working frequency of the radar sensor [12]. The radar system was equipped with two 15 dBi linearly polarized high-gain Pasternack horn antennas, the purpose of which was to increase spatial selectivity and signal-to-noise ratio at the radar system input. To extract the frequency of the respiratory and heart activity, the in-phase and quadrature signals $V_{I}(t)$ and $V_{Q}(t)$ received by the radar, sampled at a frequency of 10 kHz, are processed according to the block diagram shown in Figure 1b. Using the spectrum of the complex signal $V_{I}\left(t\right)+j\;V_{Q}(t)$, the dominant harmonic, whose complex amplitude describes, over time, an elliptical shape on the Gaussian plane, is identified. A circle centered at the origin of the Gaussian plane is obtained by taking the equalization terms of the I and Q channels and then subtracting the contribution due to static clutter. The angle described by the temporal evolution of the point along the circumference, sampled at 50 Hz, evaluated with the arctan technique [10], represents the phase variation due to the thorax movements as a result of cardiorespiratory activity. The phase spectrum, converted into a linear displacement by means of the radar phase sensitivity, allows the assessment of the cardiac and respiratory activity rates. To further increase the level of numerical accuracy, an algorithm useful to identify both the main and the higher-order harmonic signals is employed to determine the cardiorespiratory activity [10]. The signal processing, implemented in Python 3.13 language on a computer having a 3.6 GHz Intel I7 CPU, ASUSTek, Computer Inc., Taiwan, China, takes about 75 ms to extract the respiration and heart rate.

## 3. Design of the LP-CP Polarization Converter

**Figure 2:**
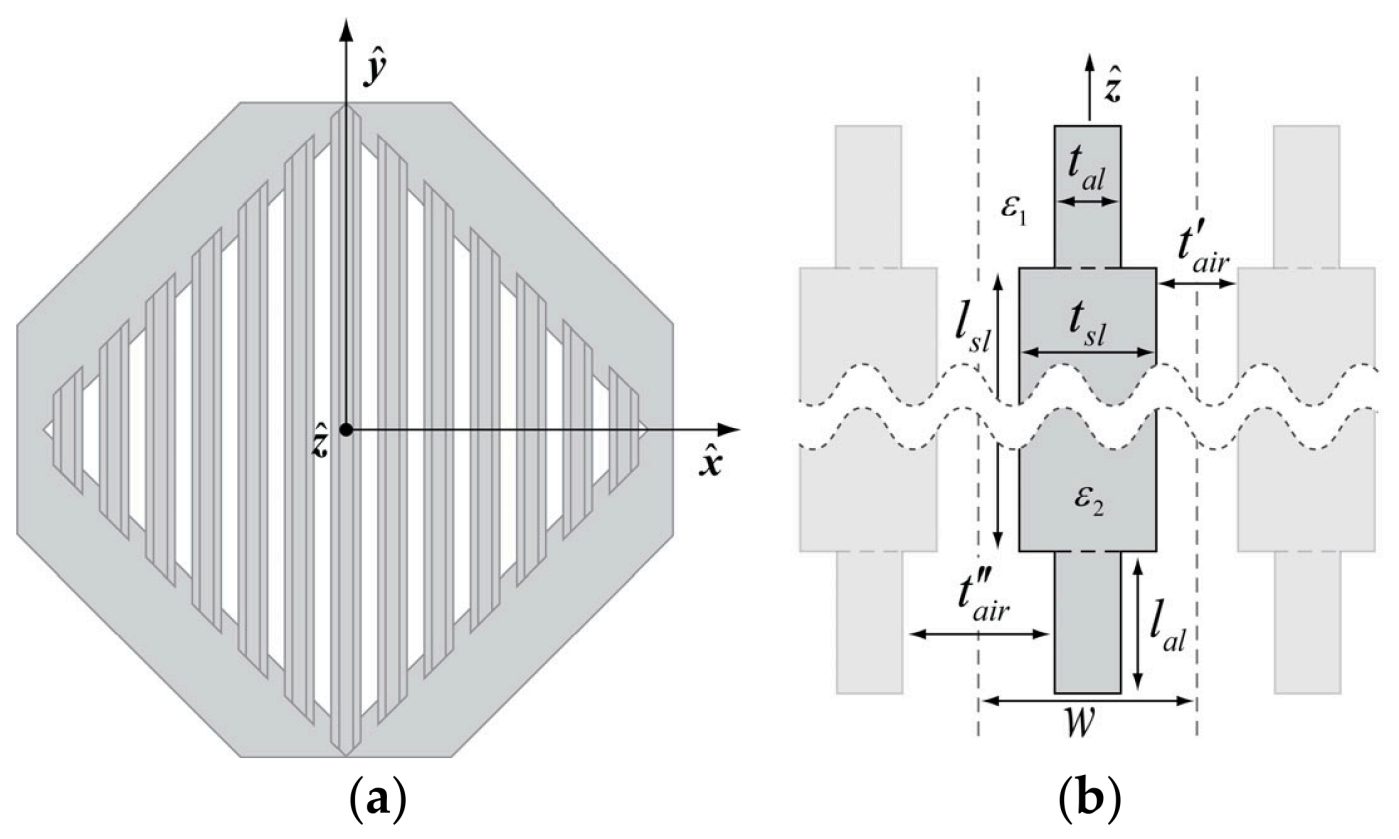
Geometrical structure of the polarization converter (**a**) and of its elementary cell (**b**). The anti-reflective layers of length $\lambda_{eff}/4$ placed at the input and output polarization converters are evident in (**b**).

**Figure 3:**
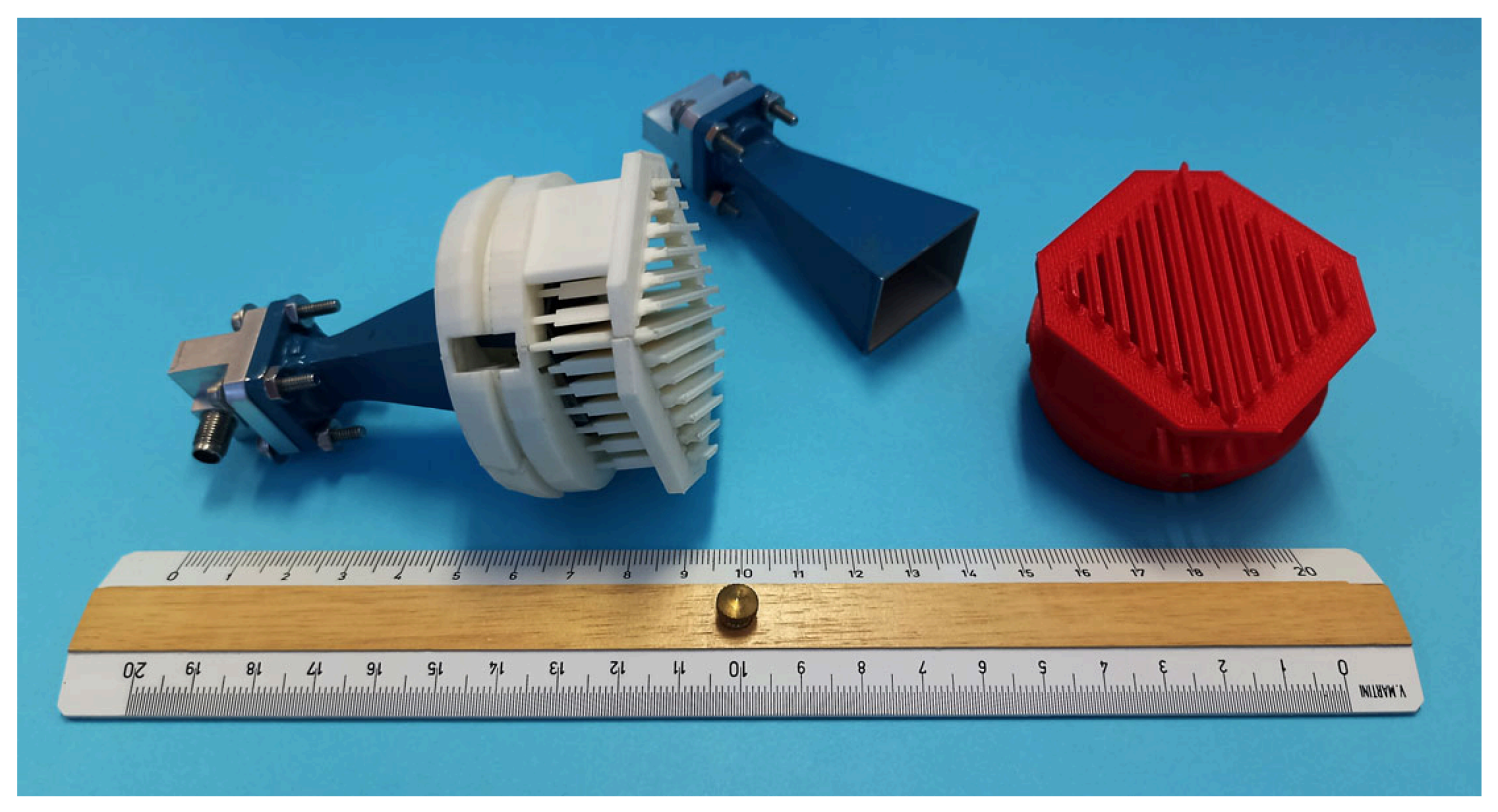
Pasternack™ 24 GHz horn antennas with and without the linear-to-circular polarization converter.

To analyze the impact of the field polarization radiated by the radar (linear/circular) on the signal-to-noise ratio level caused by environmental clutter, a specific linear-to-circular polarization converter (left-hand circular polarization (LHCP) for the Tx and right-hand circular polarization (RHCP) for the Rx antenna), easy to assemble/disassemble at the mouths of the two horn antennas, was developed. Polarization converters having opposite circular polarizations on the Tx and Rx antennas ensure high electrical isolation between the antennas, thus avoiding receiver front-end saturation, as well as rejection of even-order reflective/diffractive contributions, thereby increasing the discrimination of the useful signal from the target versus those from the environment. Particular attention was paid to the design and realization of these field converters. Among the polarization converters available in the literature, a periodic parallel dielectric plate converter was chosen because it is extremely flexible and easy to realize. To improve its performance in terms of bandwidth and polarization purity, suitable anti-reflective layers were integrated at the polarizer input and output ports (see Figure 2 and Figure 3). To this end, each dielectric plate forming the polarizer was equipped with two $\lambda_{eff}/4$ impedance matching transformers according to the indications provided in [24], with $\lambda_{eff}$ being the wavelength of the field in a medium having a dielectric permittivity equal to the geometric mean of the uniaxial tensor components related to a periodic parallel plate structure (see Figure 2).

The periodic arrangement of thin layered dielectric slabs confers on the polarization converter a uniaxial behavior [25] that can be modeled by the following equivalent dielectric permittivity tensor:

(1) $$\underline{\varepsilon }=\;\varepsilon_{x}\hat{x}\hat{x}+\varepsilon_{y}\hat{y}\hat{y}+\varepsilon_{z}\hat{z}\hat{z}$$

(see Figure 2) where $\hat{x}$, $\hat{y}$, and $\hat{z}$ are the corresponding unit-vectors, while $\varepsilon_{x},\varepsilon_{y}$, and $\varepsilon_{z}$ are the relative equivalent permittivity tensor components, whose expressions are given as follows:

(2) $$\left\{\begin{array}{l} \varepsilon_{x}\left(\xi \right)=\frac{\varepsilon_{1}\varepsilon_{2}(1+\xi )}{\varepsilon_{2}+\varepsilon_{1}\xi }+\frac{\varepsilon_{1}\varepsilon_{2}{\left(\varepsilon_{1}-\varepsilon_{2}\right)}^{2}\left(\varepsilon_{1}+\varepsilon_{2}\xi \right)}{{12\left(\varepsilon_{2}+\varepsilon_{1}\xi \right)}^{3}{\left(1+\xi \right)}^{2}}\xi^{2}\beta_{0}^{2}\;w^{2}\\ \varepsilon_{y}\left(\xi \right)=\varepsilon_{z}\left(\xi \right)=\frac{\varepsilon_{1}+\xi \varepsilon_{2}}{1+\xi }+\frac{{\left(\varepsilon_{1}-\varepsilon_{2}\right)}^{2}}{12{\left(1+\xi \right)}^{4}}\xi^{2}\beta_{0}^{2}\;w^{2} \end{array}\right.$$

where $\beta_{0}$ is the free-space wavenumber, $\varepsilon_{1}$ and $\varepsilon_{2}$ are the relative permittivities, $t_{1}$ and $t_{2}$ are the thicknesses of the slabs forming the layered structure ($t_{1}=t_{air}^{\prime }$ and $t_{air}^{\prime \prime }$, $t_{2}=t_{sl}$ and $t_{al}$ in the polarizer), while $\xi =t_{2}/t_{1}$ and $w=t_{1}+t_{2}$ is the unit cell width.

Equations (1) and (2) were used for sizing both the anti-reflective layer plates and those forming the central body of the polarization converter [oriented at 45° with respect to the antenna mouth (see Figure 3)]. The length of the polarizer (including the anti-reflection layers) was chosen so as to guarantee a 90° phase shift between the electromagnetic field components at the converter output, under the assumption of limited field reflections by the dielectric plates forming the polarizer.

The electromagnetic polarizer performances (reflection/transmission coefficients and axial ratio) were evaluated using the transmission matrices, for the *x*- and *y*- components of the incident field, of the three structures that compose it:

(3) $${\underline{T}}_{\left(x/y\right)}^{pol}=\;{\underline{T}}_{\left(x/y\right)}^{al}\;{\cdot \underline{T}}_{\left(x/y\right)}^{sl}\;\cdot {\underline{T}}_{\left(x/y\right)}^{al}$$

where ${\underline{T}}_{\left(x/y\right)}^{pol}$ is the polarizer transmission matrix, while ${\underline{T}}_{\left(x/y\right)}^{al}$ and ${\underline{T}}_{\left(x/y\right)}^{sl}$ are those modeling the anti-reflective layer and the dielectric parallel plates forming the polarizer body, respectively (see Figure 2). The matrices in (3) assume the following form

(4) T_x/yal/sl=cosϕx/yal/slj Zc,x/yal/slsinϕx/yal/slj 1Zc,x/yal/slsinϕx/yal/slcosϕx/yal/sl

with

(5) $$Z_{c,\left(x/y\right)}^{al/sl}=\frac{\eta_{0}}{\sqrt{\varepsilon_{\left(x/y\right)}\left(\xi_{al/sl}\right)}}$$

and

(6) $$\phi_{\left(x/y\right)}^{al/sl}=\beta_{0}\;\sqrt{\varepsilon_{\left(x/y\right)}\left(\xi_{al/sl}\right)}\;l_{al/sl}$$

where $\eta_{0}$ is the free-space characteristic impedance, while $l_{al/sl}$ is the length of each of the three structures forming the polarizer converter. For the converter design, a modified version of the synthesis procedure suggested in [24] was used, which consists of sizing the thicknesses of the anti-reflective plates $t_{al}$ and that of the polarizer body $t_{sl}$ after choosing the relative permittivity of the dielectric material $\varepsilon_{2}$, the unit-cell length (preferably $\beta_{0}w\ll 1$), and the total polarization converter length $l_{tot}=2l_{al}+l_{sl}$. The identification of the plates thicknesses was performed by simultaneously minimizing, using the interior point technique [26], the following cost function:

(7) $$L\left(\xi_{al},\xi_{sl}\right)={\left(\frac{\varepsilon_{x}\left(\xi_{al}\right)}{\sqrt{\varepsilon_{x}\left(\xi_{sl}\right)}}-1\right)}^{\;2}+{\left(\frac{\varepsilon_{y}\left(\xi_{al}\right)}{\sqrt{\varepsilon_{y}\left(\xi_{sl}\right)}}-1\right)}^{\;2}$$

and the phase delay constraint to obtain a linear-to-circular field conversion

(8) $$\frac{2\;\pi }{\lambda_{0}}\;\left\{{2\;l}_{al}\left[\sqrt{\varepsilon_{y}\left(\xi_{al}\right)}-\sqrt{\varepsilon_{x}\left(\xi_{al}\right)}\right]+{\;l}_{sl}\left[\sqrt{\varepsilon_{y}\left(\xi_{sl}\right)}-\sqrt{\varepsilon_{x}\left(\xi_{sl}\right)}\right]\right\}=\frac{\pi }{2}$$

with

(9) $$l_{al}=\frac{\lambda_{0}}{4\;{\left[\varepsilon_{x}\left(\xi_{al}\right)\;\varepsilon_{y}\left(\xi_{al}\right)\right]}^{\frac{1}{4}}}$$

where $\xi_{al}$ and $\xi_{sl}$ are the ratios between slab thicknesses forming the anti-reflective and polarizer body, respectively. Equations (7) and (8) ensure a 90° phase delay between the *x*- and *y*-electric field components at the converter output, thus allowing the excitation of the CP field polarization.

**Figure 4:**
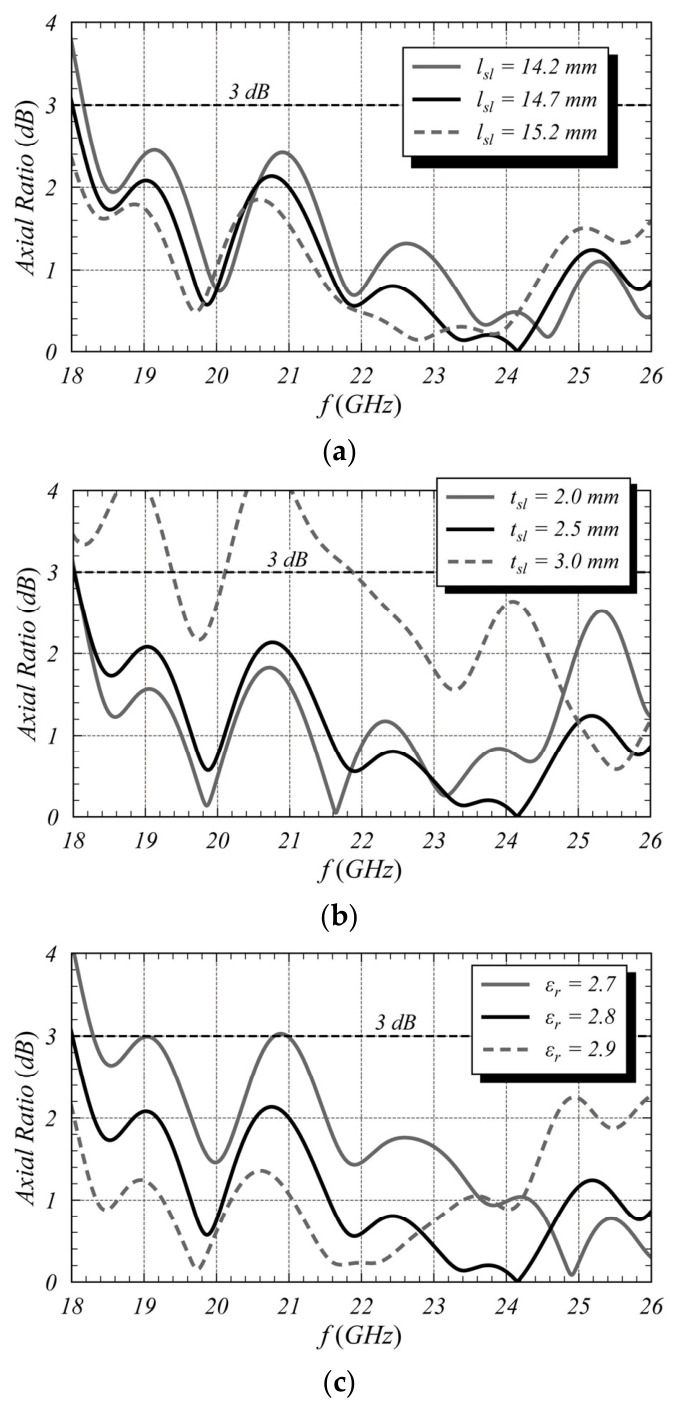
Frequency behavior of the axial ratio of the horn antennas equipped with the LP-CP polarization converter as a function of the parameters that identify: (**a**) length, (**b**) width, and (**c**) dielectric relative permittivity of the slabs forming the polarization converter.

To expedite the polarization converter design, taking into account the dispersive character due to the field focusing process in the dielectric sheets forming the polarizer, the uniaxial equivalent medium based on (1)–(2) was used for each of the three layers that make up the converter, with $\varepsilon_{1}=1$ (air) and $\varepsilon_{2}=2.8$ [Polylactic Acid (PLA) [27]] (see Table 1 for the related electrical and geometrical parameters). The PLA chosen to realize polarization converters is extremely cost-effective, having been designed as a standard material for rapid prototyping and concept modeling in engineering and consumer products. The optimization of the electromagnetic performance of the unit cell, as well as of the horn antennas equipped with the polarization converters, was carried out using the full-wave software CST Studio Suite. To achieve the LP-CP field polarization conversion in the desired frequency band (18–26 GHz), an elementary cell, describing the basic block of the converter, having a fractional bandwidth of about FBW = 43.8%, was designed. To check the final design of the polarization converter, some parametric investigations were carried out by varying the length and width of the central slabs of the converter and its dielectric permittivity, keeping the period of the elementary cell unchanged in order to limit the transverse and longitudinal converter dimensions, as well as the field reflections at the horn–converter and converter–free-space interfaces. The numerical investigation was carried out by simulating the horn antenna equipped with the polarization converter, including the dielectric frame useful for its housing at the antenna mouth, so as to include the perturbative effects due to the assembly. For the sake of brevity, Figure 4 shows the frequency behavior of the axial ratio in the 18–26 GHz band, from which it appears the optimal sizing of the polarization converter when the relative dielectric permittivity is $\varepsilon_{2}=2.8$.

After design optimization, the polarization converters were realized using a 3D-printer Raise3D E2, Stafford, TX, USA (see Figure 3) and measurements of the horn antennas equipped with their respective converters were performed. These measurements were carried out using an Agilent Technologies E8363C Vector Network Analyzer (VNA) (Agilent Technologies, Santa Clara, CA, USA) and a second calibrated horn antenna (PE9852/2F-15 of Pasternack) placed at a distance (mouth-to-mouth between antennas) of about 22 cm along the antennas boresight direction, while the axial ratio (AR) and its bandwidth were measured by rotating the calibrated antenna by 90°. Insertion losses lower than 1 dB and polarization converter permittivity of 2.7 were estimated by the performed measurements. The small discrepancy observed in the dielectric permittivity value (equal to 3.6% compared to the design value of $\varepsilon_{2}=2.8$) is caused by the 3D printing method as well as by the tolerance of the dielectric permittivity used (not for R.F. applications) for the polarizer manufacturing [27].

**Figure 5:**
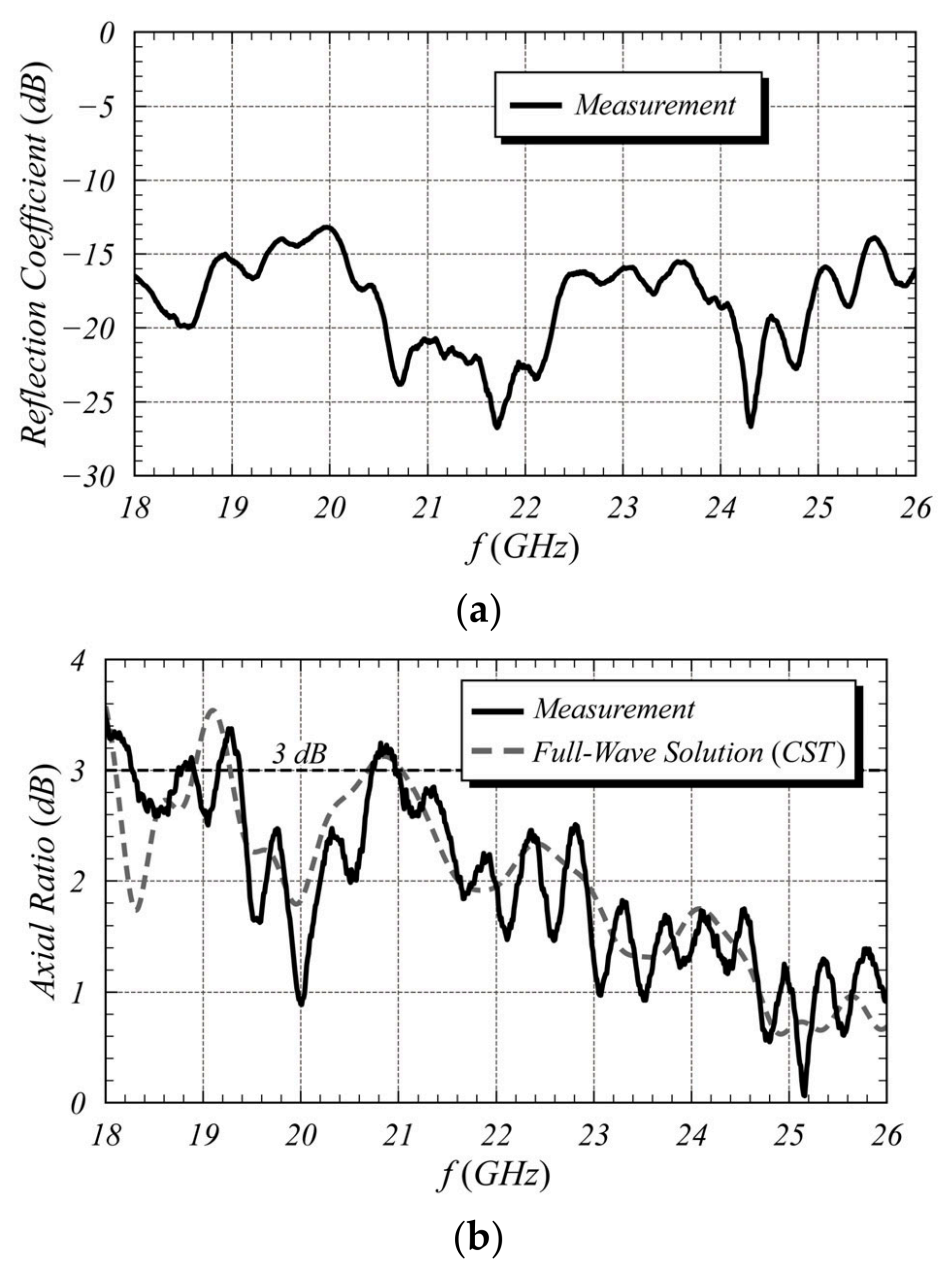
Frequency behavior of the antenna reflection coefficient (**a**) of the axial ratio (**b**), and of the horn antennas equipped with the polarization converter. A good agreement between the full-wave solution with $\varepsilon_{2}=2.7$ and the experimental measurement can be observed in the figure.

Figure 5 shows the excellent performance of the radiating system (horn with polarizer) in the 18–26 GHz frequency range in terms of antenna impedance bandwidth and of field LP-CP polarization conversion capability. These excellent performances enable the realization of simpler and more compact radar sensors than those currently used for vital signs detection [21,22,23]. For comparison purposes, Table 2 summarizes the performances of the CP radar sensors currently available in the literature.

Table 2 shows that the radar sensor presented in this paper exhibits superior performance in terms of axial ratio and electrical isolation between Tx and Rx antennas compared to other implementations proposed in the literature. Particularly significant is the improvement in environmental clutter reduction achieved by the proposed radar equipped with circularly polarized antennas compared to the ones with linearly polarized antennas.

## 4. The Electromagnetic Modeling of the Operative Scenario in the Presence of the Environmental Clutter

A GO-based model was developed to evaluate the benefits of CP polarization over LP polarization in the presence of environmental clutter. The GO model was chosen because the main beam footprint of the TX and Rx antennas falls inside the target and the vibrating metal panels, where the edge diffracted fields are subdominant with respect to the GO field [29,30]. Field contributions associated with multiple diffractions of order n are negligible since they decay as $1/\sqrt[{n}]{\beta_{0}}$ ($\beta_{0}=503\;m^{-1}$ at 24 GHz) [29].

**Figure 6:**
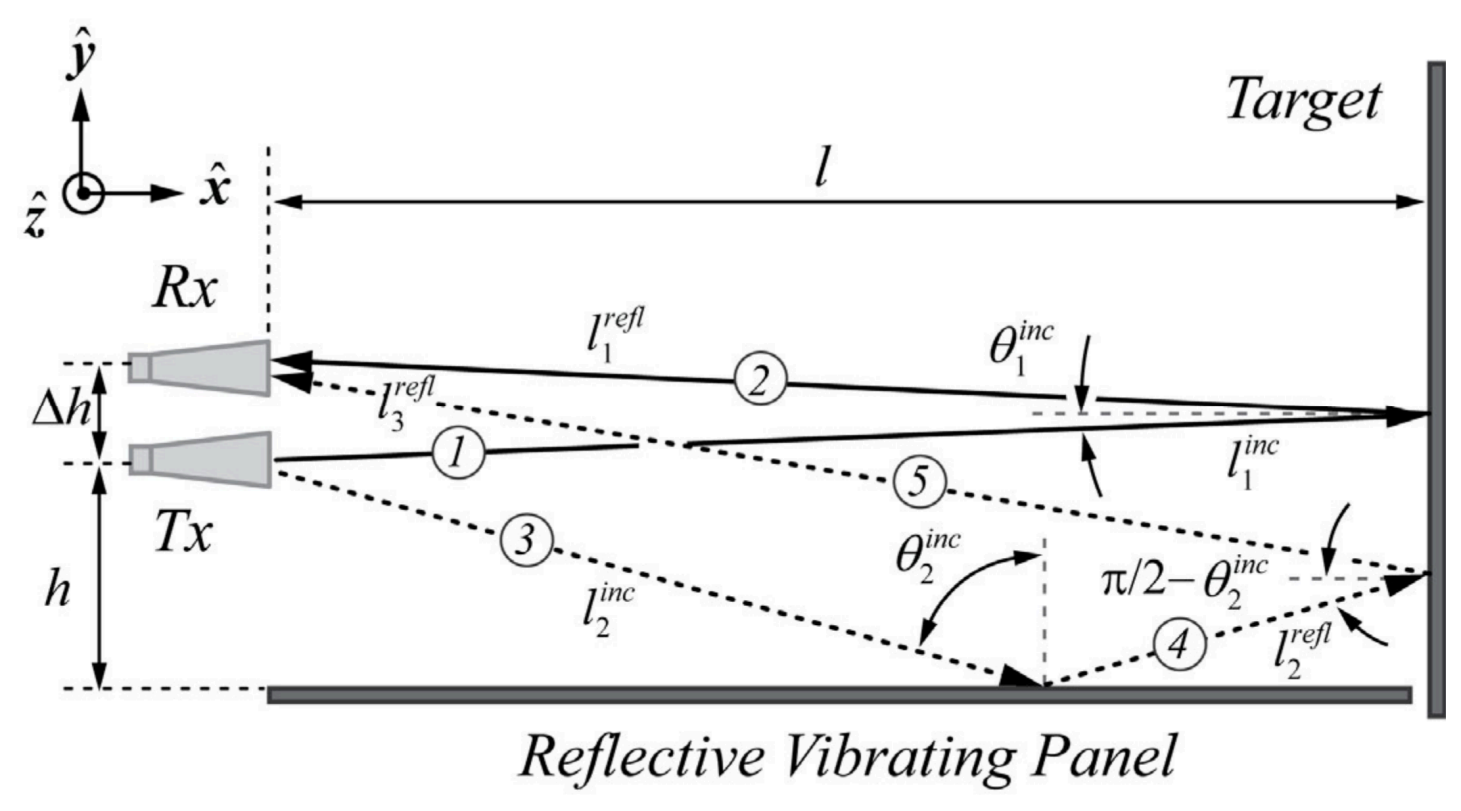
Schematic of the experimental setup adopted for the evaluation of the radar sensor robustness with respect to environmental clutter. The different electromagnetic ray paths from the Tx antenna to the Rx antenna of the radar sensor are shown in the figure.

The schematic of the radar sensor (Tx-Rx system) in the presence of the target (mimicking a human subject) and of the environmental clutter, modeled with the aid of vibrating metal panels (worst case), is shown in Figure 6. Metal panels have been proposed in the literature to simulate only human targets [23,31]. Vibrating panels, both for the simulation of the target and of environmental clutter, can be useful to radar sensor designers during experimental tests. As shown in the figure, the rays 1 and 2 represent the useful contribution (field at the target and field at the Rx), while 3, 4, and 5 represent the disturbance signal caused by environmental clutter. These fields can be modeled, according to GO, as follows:

*Field incident on the target (ray 1)*

(10) $${\underline{E}}_{1}^{inc}=\left(-sin\theta_{1}^{inc}\;\hat{x}+cos\theta_{1}^{inc}\;\hat{y}+\left\{\begin{array}{c} 0\\ j \end{array}\right\}\;\hat{z}\right)\frac{e^{-j\;\beta_{0}\;l_{1}^{inc}}}{l_{1}^{inc}}\;F\left(\theta_{1}^{inc}\right)$$

with

(11) $$\theta_{1}^{inc}=\tan^{-1}\left(\frac{∆h}{2l}\right)$$

(12) $$l_{1}^{inc}=\sqrt{l^{2}+{\left(\frac{∆h}{2}\right)}^{2}}$$

*Field incident on the Rx (ray 2)*

(13) $${\underline{E}}_{1}^{r}=\left(-sin\theta_{1}^{inc}\;\hat{x}-cos\theta_{1}^{inc}\;\hat{y}-\left\{\begin{array}{c} 0\\ j \end{array}\right\}\;\hat{z}\right)\frac{e^{-j\;\beta_{0}\;\;l_{1}}}{\;l_{1}}\;F\left(\theta_{1}^{inc}\right)$$

(14) $$l_{1}=l_{1}^{inc}+l_{1}^{refl}=\sqrt{{\left(2l\right)}^{2}+{∆h}^{2}}$$

*Incident field on the vibrating reflecting panel (ray 3)*

(15) $${\underline{E}}_{2}^{inc}=\left(sin\theta_{2}^{inc}\;\hat{x}+cos\theta_{2}^{inc}\;\hat{y}+\left\{\begin{array}{c} 0\\ j \end{array}\right\}\;\hat{z}\right)\;\frac{e^{-j\;\beta_{0}\;l_{2}^{inc}}}{l_{2}^{inc}}F\left(\frac{\pi }{2}-\theta_{2}^{inc}\right)$$

(16) $$\theta_{2}^{inc}=\tan^{-1}\left(\frac{2l}{2h+∆h}\right)$$

(17) $$l_{2}^{inc}=\frac{h}{\cos\theta_{2}^{inc}}$$

*Field incident on the target (ray 4)*

(18) $$\begin{array}{l} {\underline{E}}_{3}^{inc}=\left(-sin\theta_{2}^{inc}\;\hat{x}+cos\theta_{2}^{inc}\;\hat{y}-\left\{\begin{array}{c} 0\\ j \end{array}\right\}\;\hat{z}\right)\frac{e^{-j\;\beta_{0}\;l_{2}}}{\;l_{2}}F\left(\frac{\pi }{2}-\theta_{2}^{inc}\right) \end{array}$$

(19) $$l_{2}=l_{2}^{inc}+l_{2}^{refl}=\frac{l}{\sin\theta_{2}^{inc}}$$

*Field incident on the Rx (ray 5)*

(20) E_2r=−sinθ2inc x^−cosθ2inc y^+0j z^e−j β0 l3l3             Fπ2−θ2inc

with

(21) $$l_{3}=l_{2}^{inc}+l_{2}^{refl}+l_{3}^{refl}=\sqrt{{\left(2l\right)}^{2}+{\left(h+∆h\right)}^{2}}$$

In (10)–(21), j is the imaginary unit, *F*(*θ*) identifies the Tx antenna radiation pattern, while ∆*h* is the distance between the Tx-Rx antennas of the radar sensor. The fields and the unit vectors of the Cartesian reference system are indicated in bold, while the incidence angles and the ray paths are shown in Figure 6. Equations (10), (13), (15), (18), and (20) identify LP fields when the upper term inside the curly brackets (zero value) is used, while they describe CP fields when the lower term inside the curly brackets (imaginary unit) is considered.

As can be observed, Equations (13) and (20) describe fields incident on the polarization converter, placed on the Rx, having opposite circular polarizations (LHCP for Tx and RHCP for the Rx antenna) and therefore, the disturbance signal level (path 3, 4, 5) is drastically reduced at the output of the Rx antenna by the blocking action of the converter, as they guarantee the rejection of even-order reflected/diffracted ray contributions. This does not happen when using the LP.

**Figure 7:**
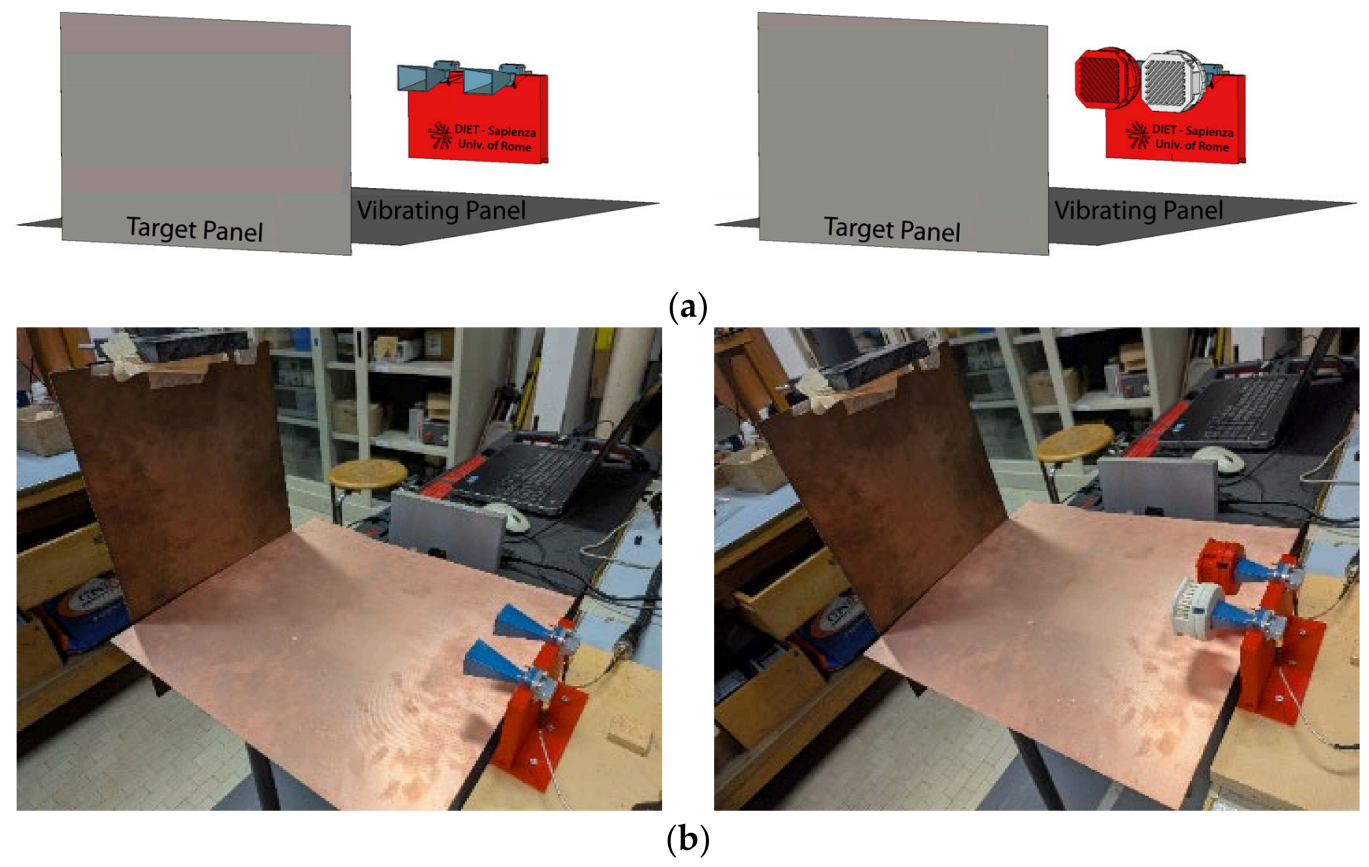
Numerical model (**a**), experimental setup (**b**) used for the validation of the GO model. Target (light gray), vibrating panel (dark gray).

**Figure 8:**
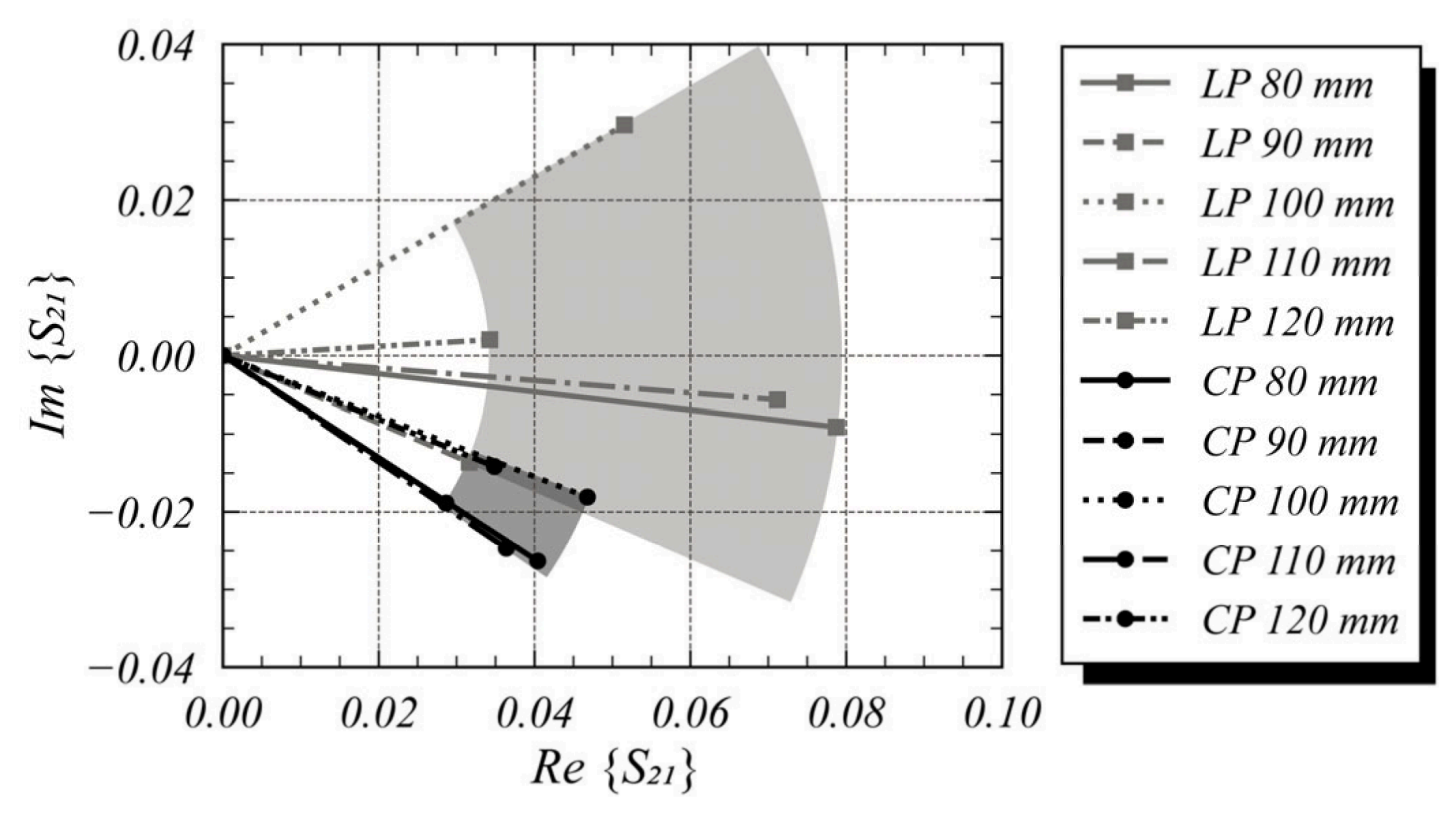
Variation in the parameter $S_{21}$ (Tx-Rx radio link) at 24 GHz obtained through full-wave numerical simulations for different positions of the horizontal disturbing panel with respect to the antenna axis. A lower variation in the $S_{21}$ parameter is obtained in the case of circular polarization (dark gray area).

The GO model has been validated by means of full-wave numerical simulations and experimental measurements. To this end, an experimental scenario, similar to that of the GO model reported in Figure 6, was analyzed. A metal panel of dimensions 45.7 cm×40.5 cm was used for the target and of dimensions 45.6 cm×30.5 cm for the vibrating panel (modeling the environmental clutter), with the latter moved by a programmable mechanical micro-linear actuator (see Figure 7). Figure 8 shows the variation in the parameter $S_{21}$ (related to the radio link Tx-Rx) at the operating frequency of 24 GHz for both polarizations and for different positions of the horizontal disturbing panel with respect to the antenna axis.

A smaller variation in the $S_{21}$ parameter is experienced by the CP polarization (see Figure 8). This indicates a lower sensitivity to the environmental clutter, since the phase variation of LP is 53.3° compared to 13.1° of CP, with a sensitivity about 4.1 times lower. The same conclusions are achieved using the experimental results (see Figure 7b), which showed a sensitivity reduction to environmental clutter by a factor of 5.3, in good agreement with the numerical results.

## 5. Radar Sensor Performance in the Presence of Environmental Clutter: Results and Discussion

The sensitivity of the radar sensor to detect human cardiac and respiratory activity in environments characterized by environmental clutter was analyzed using a programmable mechanical micro-linear actuator capable of emulating the movement of the human chest by moving a metal panel. The analysis was performed considering a target panel of 45.6 cm×30.5 cm, placed at a distance of about 45 cm from the radar antennas, and a vibrating panel of 45.7 cm×40.5 cm (see Figure 6) positioned horizontally with respect to the two antennas. The movement of the vibrating panel (simulating environmental clutter), enforcing a vibration on a metal panel mounted on a plastic pole, was monitored by a camera (having 30 frame/s) in order to evaluate its temporal behavior and the related frequency spectrum. Note that the use of mechanical emulators, useful for simulating vital activities, is not detrimental, as it can facilitate the design of radar sensors, as observed in other contexts (design and optimization of cell phone antennas) where the presence of human subjects (modeled using human phantoms [32]), strongly coupled to the antennas, is essential for the correct design of communication systems.

Three specific tests aimed at highlighting the simulated cardio-respiratory movement of the human chest, the impact of environmental clutter alone and of their combined effects were performed. The first test, concerning the identification of the cardio-respiratory chest movement, obtained by means of a programmable mechanical micro-linear actuator, was performed by forcing a mechanical displacement s(t) in accordance with data from experimental measurements performed on a person-under-test (male and female) with ECG, respiratory sensor and a 24 GHz radar [33]. For the sake of brevity, of the eleven cases considered in [33], only one example is reported below to demonstrate the suitability of vibrating panels for accurately mimicking vital activities and environmental clutter.

**Figure 9:**
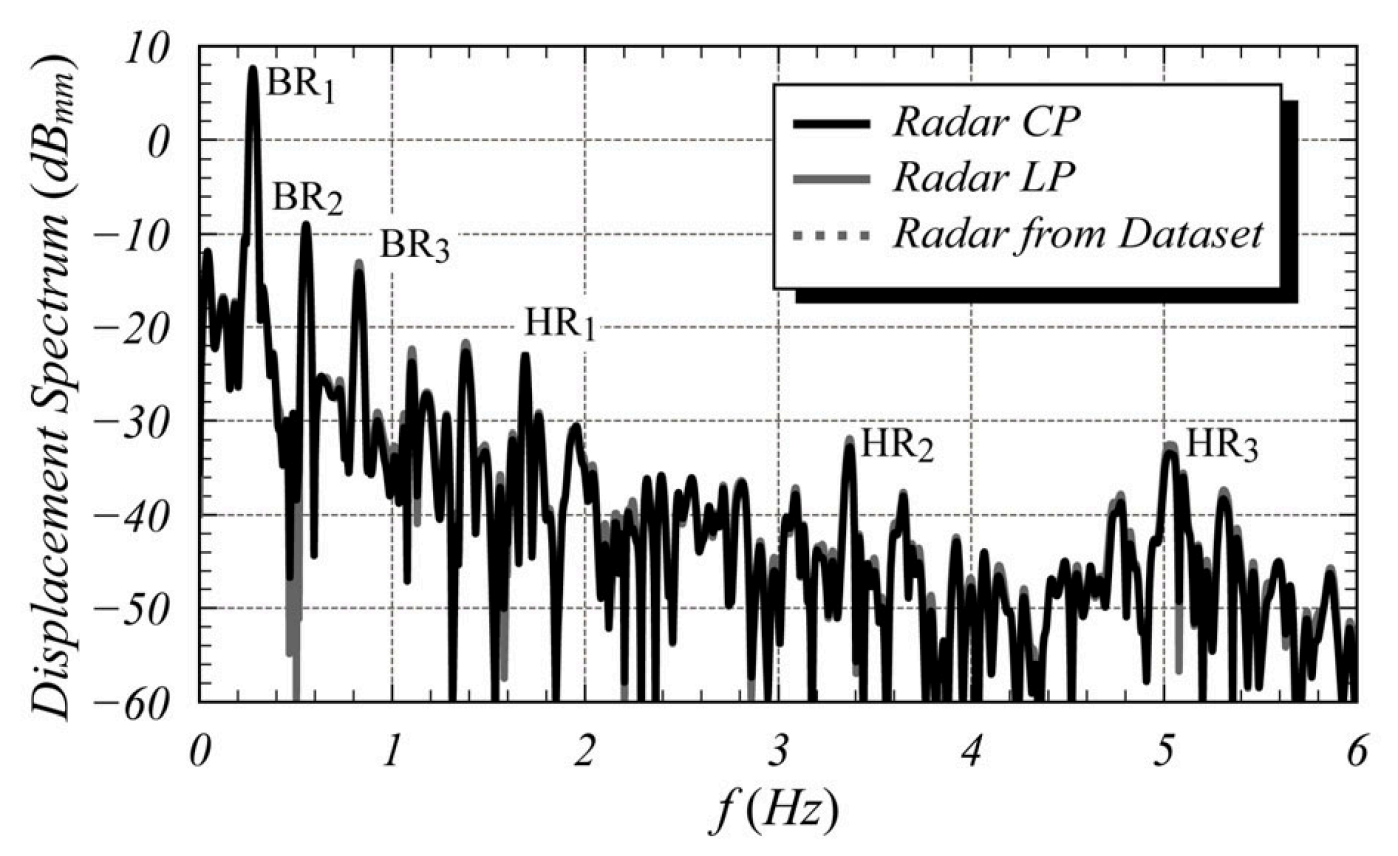
Frequency spectrum of the signal received by the radar sensor operating in LP/CP and that obtained using the data in [33] (person n° 3) in the absence of environmental interference. Note that the spectrum of the signal obtained from the dataset is not visible in the figure since it coincides with that of the LP and CP radar curves. The six harmonics characterizing the cardiorespiratory signal, not perturbed by the vibrating panel (panel in the rest position), are clearly visible compared to the background noise existing in the lab measurement environment.

The spectral behaviors of the signals detected by the radar sensor working in LP and CP, together with that of the radar signal relating to person number 3 (DATASET_2017-01-17_17-48-53_Person 3.mat) reported in [33], are shown in Figure 9. The six harmonics characterizing the cardiorespiratory signal, not perturbed by the vibrating panel (panel in rest position), are clearly visible compared to the background noise existing in the lab measurement environment. The excellent agreement between the experimental results obtained using the dataset in [33] and those obtained by the proposed radar sensor operating in LP/CP polarization (see Figure 9) demonstrates the suitability of vibrating panels for simulating vital signs.

**Figure 10:**
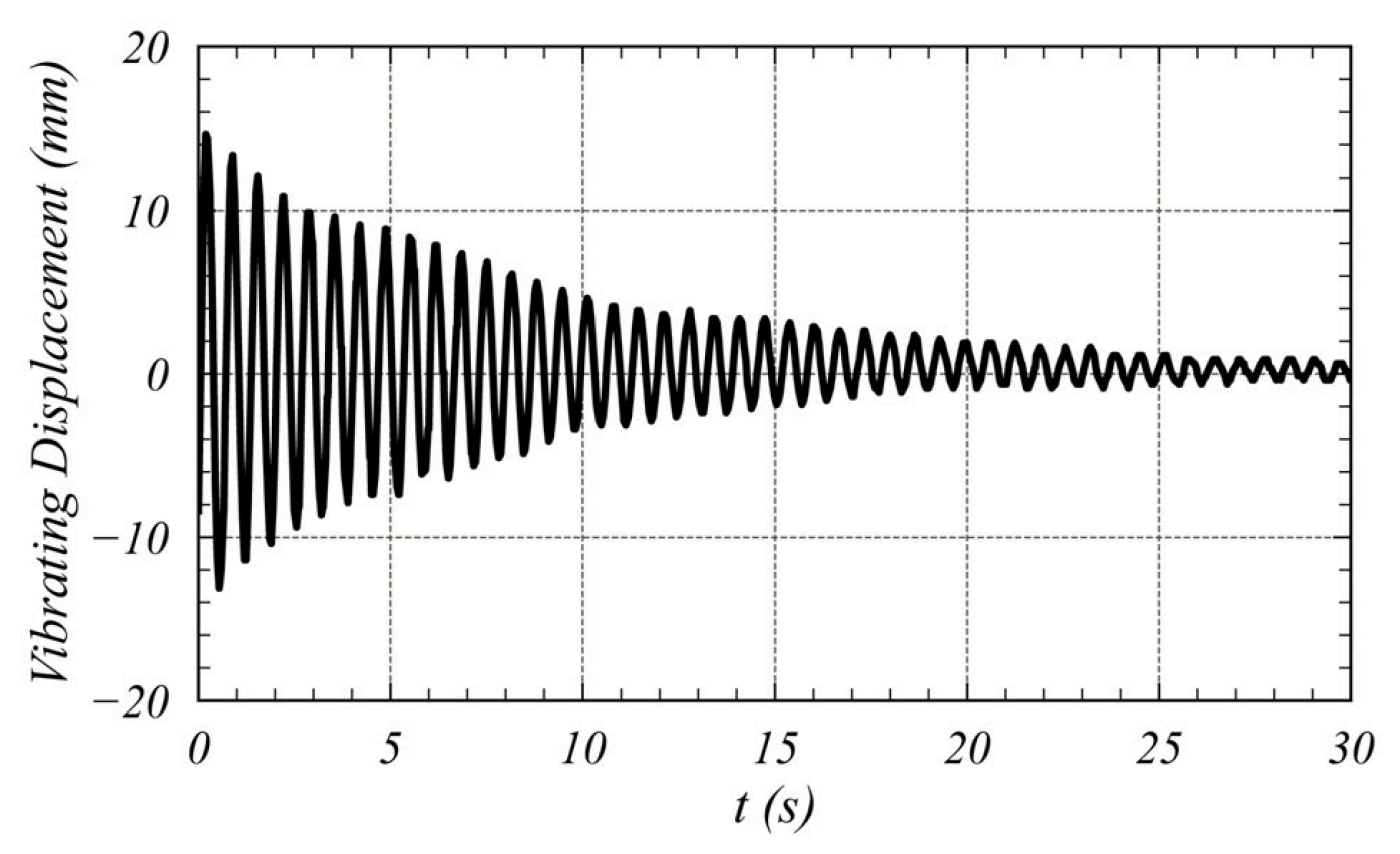
Time behavior of the vibrating panel. Time characteristics of the vibrating panel movement: initial peak-to-peak amplitude 27.1 mm, natural oscillation frequency 1.519 Hz, and damping factor $0.126\;m^{-1}$.

**Figure 11:**
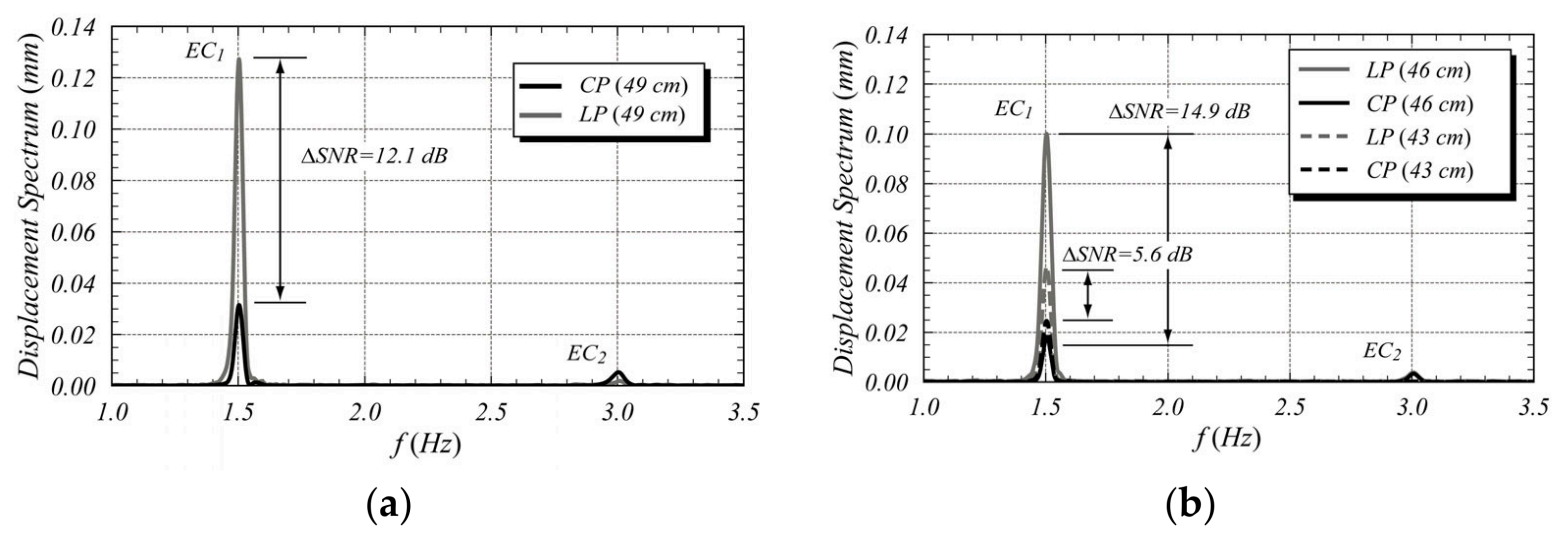
Frequency spectrum of the signal received by the radar sensor in the presence of environmental clutter (simulated by vibrating metal panels) and with the target, representative of a human subject, held stationary. Lower signal levels are detected with CP polarization. (**a**) Target placed at 49 cm from the antennas (SNR = 12.1 dB), (**b**) situations having maximum SNR (SNR = 14.9 dB) and minimum SNR (SNR = 5.6 dB) corresponding to distances of 46 cm and 43 cm from the target, respectively.

The second experimental test concerned the measurement of the sensitivity of the radar system to environmental clutter as a function of the radiated field polarization (LP or CP). During the test, the target was held stationary at 49 cm from the antennas, while the vibrating panel, placed 80 mm below the Tx-Rx antennas, was excited with an oscillation amplitude of 27.1 mm at 1.519 Hz, having a damping coefficient of about $0.126\;s^{-1}$, corresponding to a one-tenth amplitude reduction in about 18 s. The time behavior of the vibrating panel is reported in Figure 10. The frequency spectrum of the signals received by the radar sensor, operating in LP or CP, is reported in Figure 11a.

Figure 11 shows that the system using CP receives a much lower interference signal than that based on LP. In particular, the first harmonic of the signal received by the radar system operating in LP exhibits an amplitude of 0.127 mm, versus 0.032 mm for the CP case, with a sensitivity reduction to environmental clutter of about 4 times (about 12 dB), in agreement with the numerical results presented in the previous section. To establish the repeatability of the received signal level caused by the vibrating panel simulating the environmental clutter regardless of the position of the target, the same procedure described above was repeated nine times by moving the target 1 cm at a time, starting from a distance of 41 cm from the antennas. From the analysis of the data concerning the movement of the vibrating panel and the relative levels of the signal received by the radar sensor, it appears that the average peak amplitude of the movement of the vibrating panel is 27.3 mm (standard deviation 5.1 mm), the oscillation frequency is equal to 1.517 Hz (standard deviation 0.003 Hz) and the decay factor is $0.120\;m^{-1}$ (standard deviation $0.019\;m^{-1}$, while the average level of the received signal is equal to 9.6 dB (standard deviation 2.8 dB). Figure 11b also shows the spectra of the signals received by the radar in the worst and best case analyses (higher and lower received noise signal, respectively), which show the good repeatability of the experimental measurements, taking into account that the vibrating panel is operated manually.

**Figure 12:**
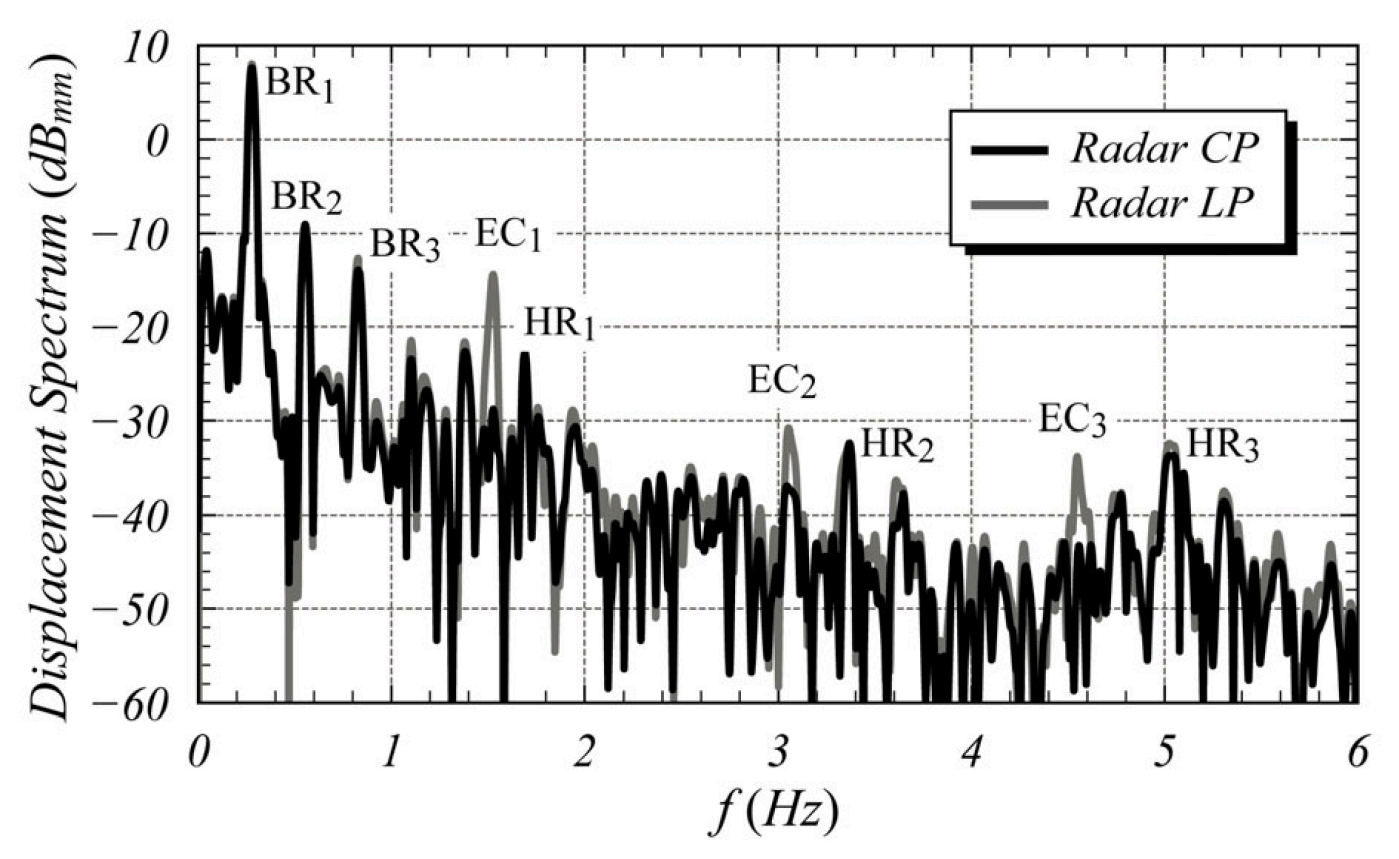
Frequency spectrum of the signal received by the radar sensor related to the cardiorespiratory activity (desired received signal) in the presence of environmental clutter (EC) for LP and CP polarization. An interfering signal level of about 12 dB higher is observed in the case of CP.

As the last investigation, two experimental tests of significant practical value were considered (regular cardiorespiratory activity, activity in the presence of cardiorespiratory arrest). Both involve the simultaneous movement of the target panel, emulating cardiorespiratory activity, and that of the disturbance panel used to mimic environmental clutter. In the first case, the movement of the panel emulating the environmental clutter was set to oscillate at a frequency close to the fundamental harmonic of the heartbeat. This specific event can be particularly unfavorable for the detection of vital heartbeat-related signals by radar sensors, as the most efficient algorithms used for heart rate measurement use both the fundamental and higher-order harmonic identification [34,35]. Figure 12 shows the frequency spectrum of the signal detected by the radar sensor when the target displacements are enforced using the dataset related to person 3 in [33] for both the LP and the CP. As can be seen, the system operating in LP also significantly reveals the level of disturbance, a level that can lead to an incorrect identification of the first harmonic of the heartbeat (the level of disturbance detected by LP is approximately 12 dB higher than that obtained with CP).

**Figure 13:**
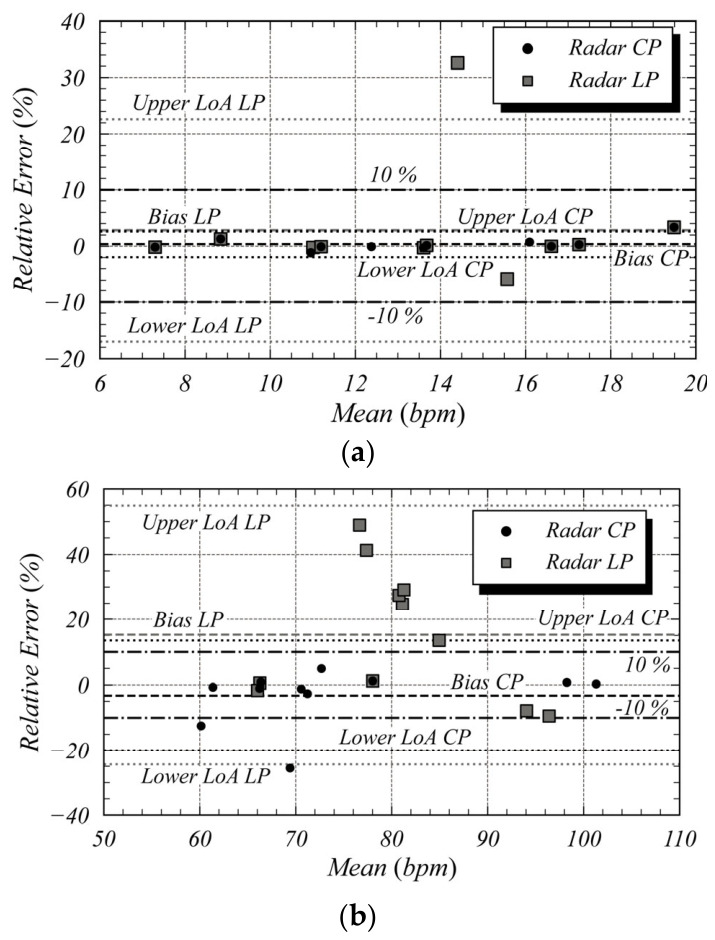
Bland–Altman diagrams of: (**a**) respiratory and (**b**) cardiac activity measurements. The 10% limits are indicated by horizontal lines to highlight the measures relating to the false detection rate.

Table 3 and Table 4 show the values of respiratory and heart rates obtained referring to the 11 human subjects in the dataset in [33]. The data tables, together with those from the ECG and the respiratory sensor, were used to realize the Bland–Altman diagrams [36] shown in Figure 13. From the analysis of the figure, it appears that the bias (average relative error) in the estimation of the respiratory rate is 2.8% for LP and 0.4% for CP, while for the heart rate, it is 15.3% for LP and −3.25% for CP. This result highlights that the mean relative error is greater in the LP case, which is strongly influenced by clutter noise, especially for the heart rate estimation. Further, the width of the Limits of Agreement (LoA width) is 39.7% for LP and 4.6% for CP for the respiration rate estimation, and 79.3% for LP and 33.7% for CP in the case of heart rate estimation. It is also evident from the LoA widths that the rate estimates performed in LP show greater measurement dispersion than those in CP. Furthermore, in accordance with the reference accuracy limits of 10% dictated in [37] for medical detecting rate devices, the false detection rate (false detections indicated on a gray background in Table 3 and Table 4) relating to the respiratory and cardiac activity is, for CP, 0/11 (1/11 for LP) and 2/11 (6/11 for LP), respectively. Finally, the SNR improvement with respect to LP is about 12 dB (with a standard deviation less than 1 dB) for both respiratory rate and heart rate. The SNR is evaluated as follows:

(22) $${\left.SNR\right|}_{dB}=10{log}_{10}\left(\frac{P_{S}}{P_{N}}\right)$$

with

(23) $$P_{S}=\frac{1}{2}\sum_{n=1}^{3}{\left|A_{S_{n}}\right|}^{2}$$

and

(24) $$P_{N}=\frac{1}{2}\frac{\sum_{m=1}^{N_{p}}\sum_{n=1}^{3}{\left|{A_{C}}_{m_{n}}\right|}^{2}}{N_{p}}$$

where $A_{S_{n}}$ are the amplitudes of the first three harmonics of the respiratory or cardiac spectrum, while ${A_{C}}_{m_{n}}$ denote the amplitudes of the first three clutter noise harmonics (*n* = 1, 2, 3) for all the considered cases ($m=1,2\ldots N_{p}$, where $N_{p}=11$). As a result of the motion enforced on the disturbing panel, the clutter harmonic frequencies are multiples of the fundamental oscillation frequency of 1.517 Hz.

Both Figure 12 and Table 3 and Table 4 show that the clutter level received by the LP radar is always higher than that received by the radar operating in CP. As a result, a clear improvement in SNR and a strong limitation of the false detection rate is observed when operating with CP radar sensors. Note that for all the considered cases, the clutter noise falls within the heartbeat signal band (0.7–2.5 Hz) and therefore cannot be filtered a priori using a software technique, such as, for example, that based on the Moving Target Indicator reported in [38], without also risking canceling the useful signal. Therefore, for these purposes, it is preferable to filter the noise employing high-gain antennas equipped with a field polarization converter.

Further investigations were conducted to identify the ability to detect vital signals in the presence of a second vibrating panel (positioned at about 20 cm from the antenna and in front of the first vibrating panel oscillating at a frequency of approximately 1 Hz), the radar detection sensitivity with respect to the orientation angle of the target with respect to the main radar beam, as well as that concerning the target covered by a fabric simulating the clothing of the subject under test. While the results concerning the presence of a second oscillating panel and those of the target covered by a cloth are substantially coincident with the corresponding case obtained with a single vibrating panel, those relating to the variation in the incidence angle show the improvement of the CP polarization with respect to the LP even if the SNR tends to worsen as the orientation angle of the target with respect to the main radar beam increases, as can be seen from the SNR values reported in Table 5.

Finally, in the second test (temporary cardiorespiratory arrest), a phase of respiratory apnea was simulated during which a temporary cardiac arrest lasting approximately 12 s occurs between two phases of regular heartbeat. For this particularly critical hospital condition, not included in the database in [33], a displacement $s\left(t\right)$ composed of six harmonic contributions, three for the heartbeat and three for the respiratory activity [11], was employed, whose expression is given by

(25) $$s\left(t\right)=\sum_{n=1}^{3}A_{Br_{n}}sin\left(2\pi \;n\;f_{Br}t+\phi_{Br_{n}}\right)+\sum_{m=1}^{3}A_{Hr_{m}}sin\left(2\pi \;m\;f_{Hr}t+\phi_{Hr_{m}}\right)\;\;\;\;\;\;\;\;\;$$

where $A_{Br_{n}}$, $A_{Hr_{m}}$ and $\phi_{Br_{n}}$, $\phi_{Hr_{m}}$ are the amplitudes and phases of respiratory/cardiac activity, respectively (see values in Table 6). The fundamental frequencies of the two chest movements describing vital activity (breathing and heartbeat) are $f_{Br}=0.167\;Hz$ and $f_{Hr}=1.36\;Hz$, corresponding to 10.0 bpm and 81.6 bpm, respectively.

**Figure 14:**
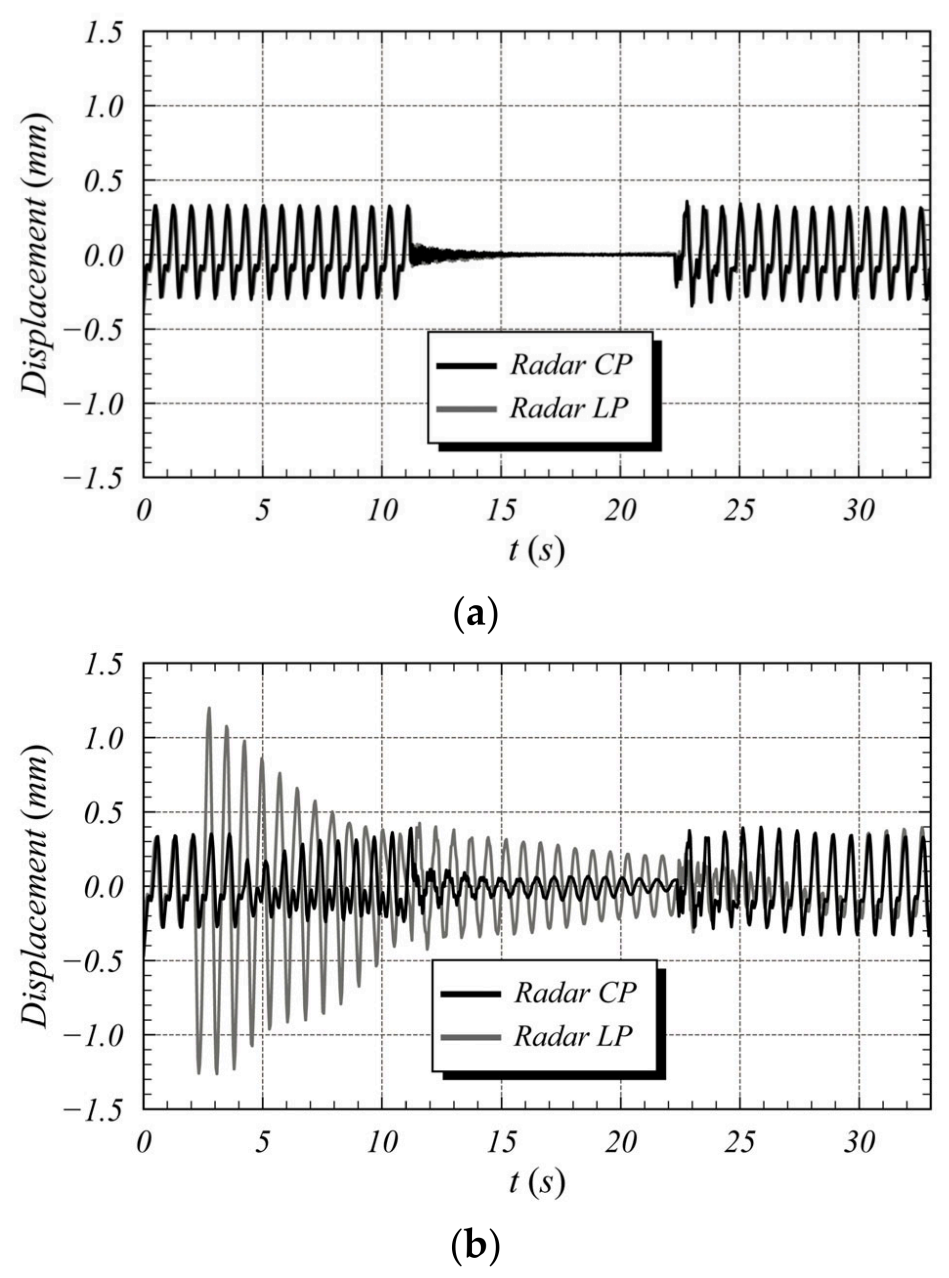
Signal behavior detected by the radar sensor when a temporary cardiac arrest is simulated. The signal was detected using the LP and CP polarization techniques, in the absence (**a**) and presence (**b**) of disturbance (emulating environmental clutter).

Figure 14a shows the temporal behavior of the movement detected by the radar sensor operating both in LP and in CP, in the absence of disturbance (environmental clutter), from which the cardiac arrest phase under apnea condition [obtained neglecting the first summation in (25)] can be seen.

**Figure 15:**
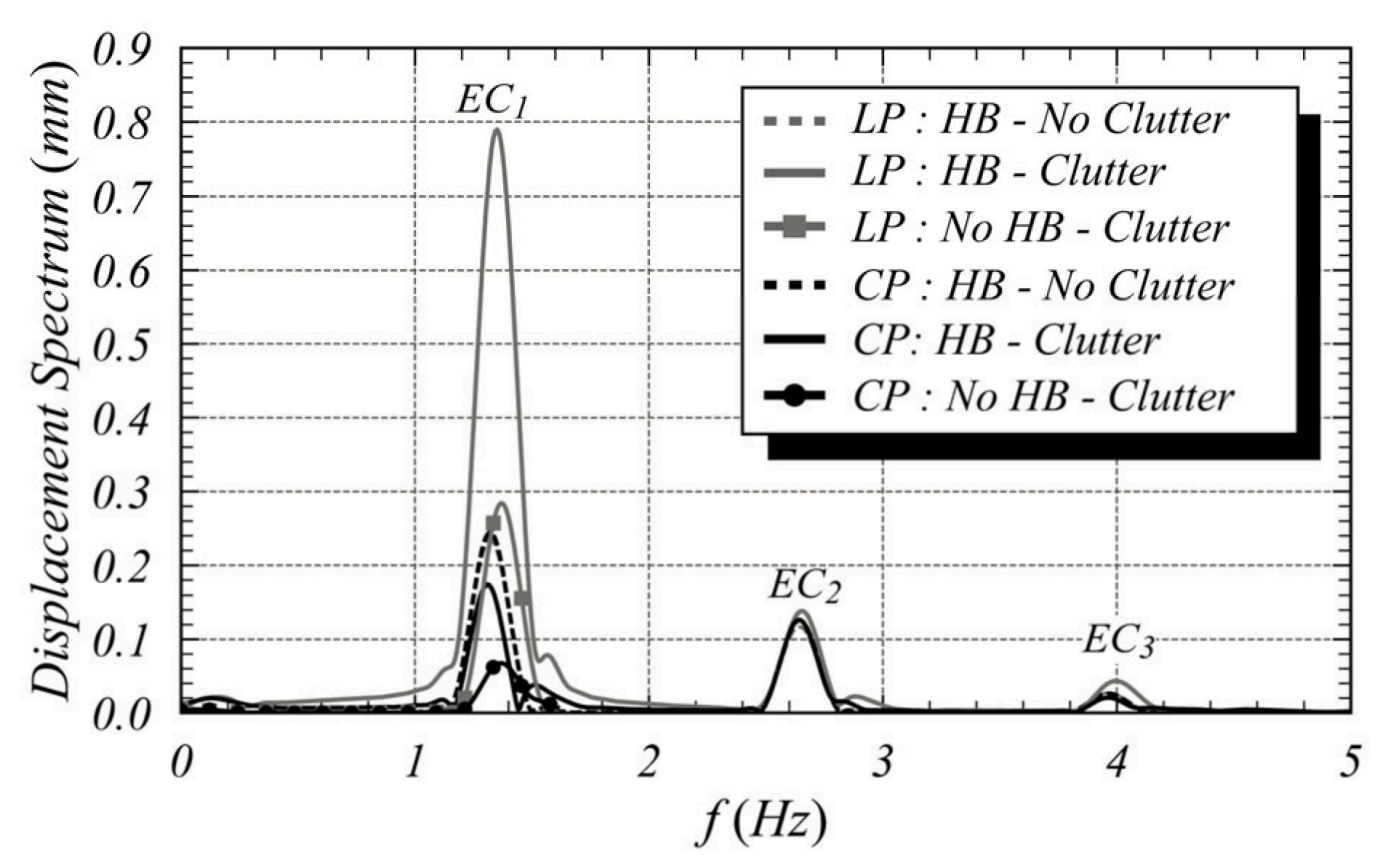
Frequency spectrum of the signal received by the radar sensor in the presence of heartbeat or cardiac arrest and environmental clutter for LP and CP polarization.

Figure 14b shows the signal detected in the presence of environmental clutter. As can be seen, CP polarization in the presence of environmental clutter allows for a clear distinction between the regular heartbeat phase and the cardiac arrest phase, while LP is unable to determine whether the patient has suffered a significant cardiac arrest. A similar conclusion can be drawn by observing the spectral profile shown in Figure 15, from which it appears that when environmental clutter and cardiac arrest occur, there is a clear difference between the spectral level detected by the radar sensor operating in LP compared to that in CP. It is evident that accurate measurement of vital signs can significantly increase the chances of survival of patients with serious illnesses undergoing remote instrumental monitoring.

## 6. Conclusions

A 24 GHz radar, operating in linear/circular polarization (LP/CP), useful for remote monitoring of vital signs (cardiac and respiratory activity), has been used to analyze the vital signs detection performance in the presence of environmental clutter. The radar system, making use of horn antennas, required the development of suitable detachable LP-CP polarization converters (LHCP for Tx and RHCP for Rx antenna) useful for improving electrical isolation between Tx and Rx radar antennas and for evaluating the robustness of circular polarization with respect to environmental clutter. The converter sizing needed the development of a periodic dielectric plate model loaded with suitable anti-reflective layers useful for limiting the field reflections at the polarizer–air interfaces, thus allowing for better sensitivity in measuring useful signals compared to disturbing ones. The design of the CP polarization converters was facilitated by the use of an equivalent uniaxial medium having dispersive behavior. These polarization converters have made it possible to simplify and compact the radar sensor structure compared to that of the CP radar systems currently used for vital signs detection. Numerical and experimental investigations (based on datasets available in the literature or carried out using vibrating panels) have highlighted the role of environmental clutter in affecting the correct detection of cardiac rhythm, compared to respiratory rhythm, since the corresponding respiratory signals are at least one order of magnitude higher than those produced by cardiac activity. The developed GO model demonstrated the advantage in the vital signs detection capability of radar sensors operating in CP with respect to those working in LP. The experimental results obtained using the dataset and the disturbing panels showed a clear improvement in SNR and a strong limitation of the false detection rate when the radar sensor operates in CP. In particular, the average SNR increases by 12 dB and consequently, the heartbeat false detection rate decreases from 6/11 for LP to 2/11 for CP. Experimental tests carried out to monitor cardiac activity in critical hospital conditions (temporary cardiac arrest), simulated by means of electromechanical systems, have shown that CP allows for detecting this critical condition in the monitored subject, highlighting the robustness of circular polarization in extracting vital signals in the presence of environmental clutter. In conclusion, the investigations conducted using experimental datasets available in the literature or using mechanical actuators have demonstrated the robustness of the CP radar sensor in detecting vital activity in subjects wearing clothing or tilted at about ±20° with respect to the radar main beam. Future directions toward the development of novel radar sensors for vital signs detection could focus on the design of novel CP reconfigurable beam antenna systems useful for better tracking of the human subject during its movements.

## Figures and Tables

**Table 1:** Main Electrical and Geometric Parameters of the Polarization Converter.

Parameters	Value
$t_{sl}$	2.5 mm
$t_{al}$	1.2 mm
$l_{sl}$	14.7 mm
$l_{al}$	2.6 mm
w	4 mm
$\varepsilon_{r}$	2.8

**Table 2:** Performance comparison between cp radar sensors for vital signs detection available in the literature.

	*f* (GHz)	Antenna Type	Gain (dB)	Axial Ratio (dB)	Isolation (dB)	SNR Improvement LP-CP (dB)	Phase Demodulation Technique
[21]	3–10	Sequentially rotated Vivaldi antenna array	NA	<4	NA	NA	UWB BP filtering
[22]	2.4	Orthogonally excited square patch array	3	<3	<30	NA	Doppler FFT with LP-BP filtering
[23]	5.8	Sequentially rotated square patch array	6	<1.1	<60	5.6	Doppler MDACM [28]
This work	24.125	Horn antennas with LP-CP field polarization converter	15.4	<1.8	<65	12	SFMCW FFT

**Table 3:** Performance comparison between LP/CP radar sensors for breath rate detection *.

Person	Resp. Sens. in [33] (bpm)	Our LP Radar (bpm)	R.E. LP-Sens. (%)	SNR LP (dB)	Our CP Radar (bpm)	R.E. CP-Sensor (%)	SNR CP (dB)
1	12.4	16.4	32.3	11.6	12.4	0.0	24.8
2	7.30	7.29	−0.1	22.2	7.29	−0.1	34.7
3	16.6	16.6	0.0	24.2	16.6	0.0	35.2
4	19.2	19.8	3.1	20.5	19.8	3.1	31.6
5	11.0	11.0	0.0	19.8	10.9	−0.9	30.7
6	11.2	11.2	0.0	23.2	11.2	0.0	34.9
7	8.78	8.89	1.3	18.6	8.89	1.3	31.6
8	17.2	17.3	0.6	21.7	17.3	0.6	33.4
9	16.0	15.1	−5.6	20.8	16.2	1.3	32.6
10	13.6	13.6	0.0	21.2	13.6	0.0	33.6
11	13.7	13.7	0.0	19.2	13.7	0.0	31.9

(*) The gray background indicates false detection rates.

**Table 4:** Performance comparison between LP/CP radar sensors for heart rate detection *.

Person	ECG Sens. in [33] (bpm)	Our LP Radar (bpm)	R.E. LP-Sens. (%)	SNR LP (dB)	Our CP Radar (bpm)	R.E. CP-Sensor (%)	SNR CP (dB)
1	72.2	90.0	24.7	−5.67	70.3	−2.6	6.10
2	71.0	90.5	27.5	−2.83	70.1	−1.3	8.18
3	101	91.6	−9.3	−5.32	101	0.0	5.24
4	77.6	78.5	1.2	−2.62	78.5	1.2	8.51
5	64.1	90.6	41.3	−6.48	56.1	−12.5	4.58
6	79.6	90.3	13.4	−9.05	59.2	−25.6	2.64
7	61.6	91.7	48.9	−1.56	61.1	−0.8	10.1
8	66.1	66.4	0.5	−0.44	66.6	0.8	12.3
9	66.6	65.5	−1.7	4.37	65.9	−1.1	16.6
10	97.9	90.2	−7.9	−8.61	98.6	0.7	5.27
11	70.9	91.6	29.2	−7.22	74.4	4.9	5.06

(*) The gray background indicates false detection rates.

**Table 5:** Performance comparison, for specific test cases, between LP/CP radar sensors for heart rate detection.

Tilted Angle /Situation	ECG Sens. in [33] (bpm)	Our LP Radar (bpm)	R.E. LP-Sens. (%)	SNR LP (dB)	Our CP Radar (bpm)	R.E. CP-Sensor (%)	SNR CP (dB)
20°	101	90.2	−10.7	−5.25	58.1	−42.5	1.17
10°	101	88.9	−12	−9.24	101	0	6.27
0°	101	91.6	−9.31	−5.32	101	0	5.24
−10°	101	89.3	−11.6	−14.1	101	0	8.37
−20°	101	89.1	−11.8	−16.9	89.4	−11.5	−11.9
Cloth (Pers. 3)	101	89.8	−11.1	−1.41	101	0	5.93
2 Panels (Pers. 3)	101	89.6	−11.3	−1.49	101	0	12.9

**Table 6:** Parameters Describing Respiratory and Cardiac Activities.

Harmonic Order	Peak Amplitude (mm)	Phase (rad)
1st Breathing	10	0
2nd Breathing	2	0.785
3rd Breathing	1	0.524
1st Heart	0.26	−1.91
2nd Heart	0.13	−1.53
3rd Heart	0.026	0.93

## Data Availability

The raw data supporting the conclusions of this article will be made available by the authors on request.

## Abbreviations

The following abbreviations are used in this manuscript:

ADC	Analog-to-Digital Converter
AR	Axial Ratio
CP	Circular Polarization
FBW	Fractional Bandwidth
FMCW	Frequency Modulated Continuous Wave
GO	Geometric Optics
LP	Linear Polarization
NI	National Instruments
MIMO	Multiple Input Multiple Output
PLA	Polylactic Acid
PLL	Phase-Locked Loop
RF	Radio Frequency
RX	Receiver
SFCW	Single Frequency Continuous Wave
SFMCW	Sine Frequency Modulation Continuous Wave
SIMO	Single Input Multiple Output
SISO	Single Input Single Output
SNR	Signal Noise Ratio
TX	Transceiver
VNA	Vector Network Analyzer
